# Acrylated Composite Hydrogel Preparation and Adsorption Kinetics of Methylene Blue

**DOI:** 10.3390/molecules22111824

**Published:** 2017-10-26

**Authors:** Jinpeng Wang, Xiaobing Meng, Zheng Yuan, Yaoqi Tian, Yuxiang Bai, Zhengyu Jin

**Affiliations:** 1 State Key Laboratory of Food Science and Technology, Jiangnan University, Wuxi 214122, Jiangsu, China; wangjinpeng1984@126.com (X.M.); happygirlwjp@gmail.com (Z.Y.); yqtian@jiangnan.edu.cn (Y.T.); ybai@jiangnan.edu.cn (Y.B.); fpcenter@jiangnan.edu.cn (Z.J.); 2 School of Food Science and Technology, Jiangnan University, Wuxi 214122, Jiangsu, China; 3 Synergetic Innovation Center of Food Safety and Nutrition, Jiangnan University, Wuxi 214122, Jiangsu, China

**Keywords:** cyclodextrin hydrogel, microcrystalline cellulose, gel property, acrylic acid, adsorption

## Abstract

By using cyclodextrin (α-CD) self-assembly into a hydrogel with the triblock copolymer Pluronic F127, nanomicrocrystalline cellulose was introduced into a gel system to form a composite CNC-β-CD/α-CD/Pluronic F127 hydrogel (CCH). CCH was modified further by grafting acrylic acid to form a novel acrylated composite hydrogel (ACH). The swelling degree of ACH was 156 g/g. Adsorption isotherms show that the adsorption process for methylene blue proximity fitted the Freundlich model. The adsorption kinetics showed that ACH followed a quasi-second-order kinetic model. Methylene blue desorption showed that ACH was a temperature- and pH-dependent gel. Repeated adsorption and desorption experiments were carried out three times, and the removal rate of methylene blue at 75 mg/L was still 70.1%.

## 1. Introduction

In recent years, cyclodextrin (CD) supramolecular hydrogels have become a popular research topic. The basic characteristics of general hydrogels, such as biocompatibility, a high water content, drug-free delivery, and other characteristics of unique self-assembly and molecular recognition have been studied to provide intelligent CD supramolecular hydrogels that respond to shear, pH, temperature, light, and other stimuli [1]. Li et al. prepared α-CD/ polyethyleneglycol (PEG) supramolecular hydrogels for the first time at room temperature and found that the gel was temperature-responsive [2]. Such a supramolecular hydrogel preparation process is simple, mild, and exhibits a temperature response, which can be used as a drug-carrier injection gel and tissue-engineering scaffold. Zhao et al. prepared a hydrogel, which has the capacity for light stimulation, and β-CD that was modified with deoxycholic acid could encapsulate the different structures of azobenzene that were grafted on polyacrylic acid selectively [3]. Supramolecular hydrogel gel–sol transition was achieved by ultraviolet and visible-light irradiation [4]. Kretschmann et al. prepared a series of gels by the self-assembly of β-CD dimers with poly(*N*-isopropylacrylamide) to form a temperature-responsive gel [5]. Manakker et al. studied the gel by modifying cholesterol with PEG into a linear, a four-arm star, and an eight-arm star structure, and mixed the product with β-CD to obtain a supramolecular hydrogel [6,7]. The eight-arm star hydrogel exhibited the largest mechanical strength. Choi et al. grafted β-CD to a polylysine side chain to form a gel with 3-trimethylsilylpropionic acid) [8]. This gel system responded to a pH stimulus. Weickenmeier et al. made CD that was grafted to the side chain of polymaleic acid, and formed a complex with *p*-*tert*-butylbenzene to yield a gel system [9]. Gref et al. prepared β-CD polymers by crosslinking and formed supramolecular nanogels with dextran that was grafted with lauryl [10,11].

Stimuli-responsive cyclodextrin hydrogels have been recognized as promising carriers that may enhance the therapeutic efficacy and minimize side effects. They have been used as a drug, as a gene-release vector, and in biomedical tissue-engineering materials [12]. Although many researchers have attempted to make CD supermolecular hydrogels, the search for improved mechanical properties and greater intelligence to suit different applications are drivers for developing CD supramolecular hydrogels further. Nanoparticle addition into a gel system provides much stronger mechanical properties. Wang et al. attached carbon nanotubes into the block copolymer poly(ethylene oxide)-*b*-poly(propylene oxide)-*b*-poly(ethylene oxide) (PEO-PPO-PEO) gel system and found that carbon-nanotube addition could accelerate gel formation [13]. Ma et al. added Fe_3_O_4_ nanoparticles into the alpha cyclodextrin/poly(ε-caprolactone)/polyethyleneglycol/poly(ε-caprolactone)(α-CD/PCL-PEG-PCL) gel system to prepare a supramolecular hydrogel with magnetic properties [14]. Those applied nanoparticles inspired new ideas to use nanomicrocrystalline cellulose (CNC) as cheaper nanoparticles for cyclodextrin hydrogel.

CNC is a pollution-free renewable natural reinforced material with a large surface area, adsorption capacity, and good mechanical properties. However, its direct application has been limited because it agglomerates. In this study, CNC was modified by grafting citric acid-β-CD, and the product was incorporated into a gel system by mixing with the copolymer Pluronic F127 and α-CD to strengthen the gel mechanically. However, the adsorption properties were unsatisfactory. The gel was then modified by grafting acrylic acid, and the modification conditions were studied. The final gel was used in methylene-blue adsorption to study the adsorption kinetics.

## 2. Results and Discussion

### 2.1. Fourier-Transform Infrared Characterization of CNC-β-CD

**Figure 1:**
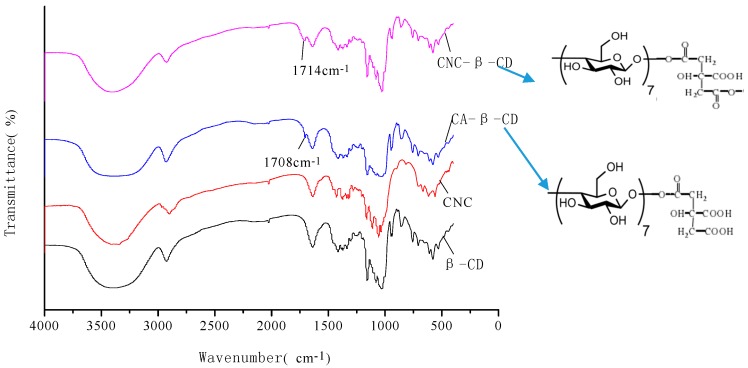
FT-IR spectra of β-CD, CNC, CA-β-CD, and CNC-β-CD.

Figure 1 shows the Fourier-transform infrared spectra of the β-CD, CNC, and CA-β-CD compared with CNC-β-CD.

The peak shape of β-CD, CNC, CA-β-CD, and CNC-β-CD is basically the same, because the structural units of both β-CD and CNC are glucose [15]. Strong and broad peaks at 3300–3500 cm^−1^ are attributed to the O–H stretching vibrations, those at 2850–2900 cm^−1^ belong to the C–H stretching vibrations, those at 1640 cm^−1^ belong to the O–H deformation vibrations and those at ~1000–1200 cm^−1^ are the C-O stretching vibrations. An intensive absorption band appeared at 1708 cm^−1^ for CA-β-CD might be the peak of the ester bond. The peak shift of absorption band at 1714 cm^−1^ for CNC-β-CD compared with 1708 cm^−1^ for CA-β-CD confirmed that the neighboring groups differed for the β-CD graft with CNC and CA. These results indicate that the CD is linked to the CNC via an ester bond.

### 2.2. ACH Preparation

After CNC-β-CD had been prepared, CCH was formed by mixing CNC-β-CD, α-CD, Pluronic F127, and water. The freeze-dried product was applied for further grafting of acrylic acid. This process undergoes three steps of chain initiation, chain growth, and chain termination [16]. The effects of acrylic–acid neutralization, temperature, and composite gel content on gel swelling were investigated.

#### 2.2.1. Effect of Neutralization Degree of Acrylic Acid on Hydrogel-Swelling

Neutralization occurs between acrylic acid and sodium hydroxide. A low neutralization degree implies the existence of a high content of acrylic acid in the system, which promotes excessive polymer cross-linking, and is accompanied by the cross-linking reaction of acid anhydride, which is not conducive to gel preparation [17]. Sodium hydroxide reacts with acrylic acid to form -COONa, which can be ionized to -COO-. The hydrophilicity of -COO- is better than COOH, which causes -COO- protonation. The ion concentration and osmotic pressure in the gel decreases, which decreases the final swelling degree of the gel. With an increase in neutralization degree, the content of -COO-, the repulsion between the gel, and the swelling degree in the reaction system increased, and the network structure expanded. 

**Figure 2:**
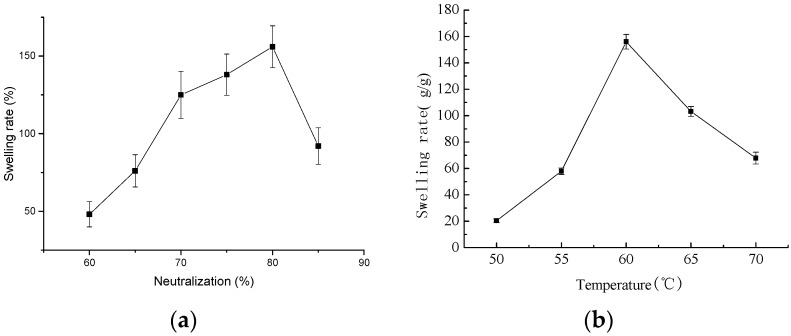

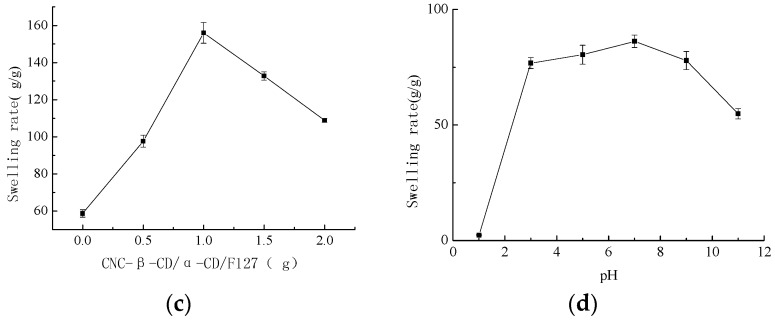
Effect of the reaction conditions on the swelling of the hydrogel. (**a**) is effect of neutralization degree on the swelling of the hydrogel, the reactions are happened following the steps as 1 g of freeze-dried CCH was weighed in 30 mL acrylic–acid solution (60%, 65%, 70%, 75%, 80%, 85%), 10 mL 0.01 g/mL *N*,*N*′-methylene bisacrylamide, and 10 mL 0.1 g/mL potassium persulfate were added to 50 mL deionized water, the mixtures were kept at 60 °C to form gels, swelling rate was calculated by Equation (1); (**b**) is effect of reaction temperature on the swelling of the hydrogel, the reactions are happened following the steps as 1 g of freeze-dried CCH was weighed in 30 mL acrylic–acid solution 80%, 10 mL 0.01 g/mL *N*,*N*′-methylenebisacrylamide, and 10 mL 0.1 g/mL potassium persulfate were added to 50 mL deionized water, the mixtures were kept at 50, 55, 60, 65, 70 °C to form gels, swelling rate was calculated by Formula (1); (**c**) is effect of weight of CCH on the swelling of the hydrogel, the reactions are happened following the steps as 0, 0.5, 1, 1.5, 2 g of freeze-dried CCH was weighed in 30 mL acrylic–acid solution 80%, 10 mL 0.01 g/mL *N*,*N*′-methylenebisacrylamide, and 10 mL 0.1 g/mL potassium persulfate were added to 50 mL deionized water, the mixtures were kept at 60 °C to form gels, swelling rate was calculated by Equation (1); (**d**) is effect of pH on the swelling of the hydrogel, the reactions are happened following the steps as 1 g of freeze-dried CCH was weighed in 30 mL acrylic–acid solution 80%, 10 mL 0.01 g/mL *N*,*N*′-methylenebisacrylamide, and 10 mL 0.1 g/mL potassium persulfate were added to 50 mL water with pH adjusted into 1, 3, 5, 7, 9, 11, the mixtures were kept at 60 °C to form gels, swelling rate was calculated by Equation (1).

When the neutralization degree exceeded 80%, the concentration of Na^+^ in the system increased, -COO- existed as -COONa, the electrostatic repulsion in the gel system decreased, and the network structure of the gel shrank. If the neutralization degree is too high, the reactivity and the reaction ratio slow down and the swelling degree decreases. The effect of neutralization on the swelling is shown in Figure 2a. The optimum neutralization degree of acrylic acid is 80% with a maximum swelling of up to 156 g/g.

#### 2.2.2. Effect of Reaction Temperature on Hydrogel Swelling

The swelling increased firstly and then decreased with an increase in reaction temperature, and it reached a maximum at a reaction temperature of 60 °C (Figure 2b). The main reason may be that a lower reaction temperature resulted in a lower reaction rate and grafting ratio. The polymer cannot form an effective three-dimensional network, so the water adsorption decreased.

As the reaction temperature increased, free radicals promoted polymerization initiation, and the gel swelled with an increase in temperature [17], which favors acrylic monomer entrance into the internal gel framework, and accelerates the graft copolymerization reaction. If the reaction temperature is too high, the graft copolymerization rate decreases because of chain termination and chain transfer. The self-polymerization reaction of acrylic acid is accompanied by these reactions, which results in a rapid uncontrolled reaction rate. The reaction heat is difficult to eliminate with time, and polymers assemble easily, which is harmful for water adsorption by the gel.

#### 2.2.3. Effect of CCH Amount on Hydrogel Swelling

With an increase in CCH addition, the swelling increased firstly and then decreased (Figure 2c). The swelling reached a maximum for 1 g CCH addition in the system. In this free-radical-polymerization reaction, CCH served as the polymer backbone. If the CCH content is less than 1 g, the acrylic–acid grafting site increases with an increase in CCH content, which favors polymerization. However, if the CCH content is too low, acrylic acid in the reaction system is not only grafted on the CCH, but also self-polymerized, which results in a poor grafting and swelling rate. If the CCH addition exceeds 1 g, the concentration of acrylic monomer decreases, and the acrylic–acid chain that linked to the same amount of CCH is reduced. This results in a short branch length and a dense gel network, which decreases the swelling degree.

### 2.3. Effect of pH on ACH Swelling

The effect of different pH on ACH swelling was investigated. The results are shown in Figure 2d. With an increase in pH, the swelling of the gel increases firstly and then decreases. The gel has a low swelling degree with low pH (pH 1), because -COOH without ionization mainly exists at low pH, the hydrogen bond that is formed between -COOH and -OH is strengthened, and the electrostatic repulsive force is weakened. The hydrogel pores were shrink, so that water molecules cannot enter into the hydrogel, which results in a low swelling degree [18,19]. The degree of swelling is kept stable in the pH range of 3 to 9. If the pH exceeds 9, the swelling is reduced for two reasons: first, the combination of Na^+^ with COO- reduces the electrostatic repulsion, which results in a decrease in swelling. Second, the CD solubility increases above pH 9, which destroys the gel in an alkaline environment.

### 2.4. Adsorption Characteristics of ACHs

Methylene blue is a heterocyclic aromatic compound. It is used most widely as a dye in the textile, printing and dyeing, leather and plastics industries, and other fields. It is also a pollution source in the effluent of the textile and other industries. Recent studies have shown that methylene blue is reducible and can be used to treat methemoglobinemia, cyanide poisoning, carbon monoxide poisoning, and Alzheimer's disease. It can also be used to test whether the adsorbent’s performance is good because of its ability to measure the adsorption capacity of adsorbents with 20–500 Å pores [20,21,22,23]. Therefore, methylene blue was chosen as the adsorption–desorption guest to characterize the adsorption and release characteristics of the gel.

#### 2.4.1. Effect of Initial Methylene-Blue Concentration on Gel Adsorption

**Figure 3:**
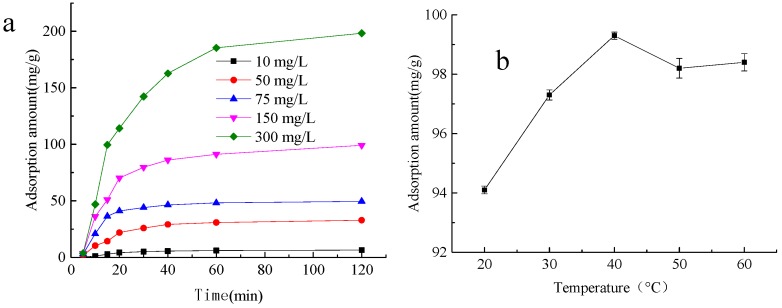

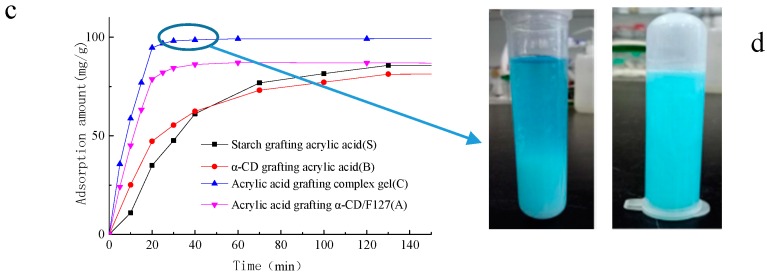
Effect of different adsorption conditions on adsorption rate of methylene blue. (**a**) is effect of adsorption time on adsorption rate of methylene blue, methylene blue solution was prepared with content of 10, 50, 75, 150, 300 mg/L, 0.1, 5 g of ACH was placed in 100 mL of methylene blue solution and incubated at 20 °C for 5, 10, 15, 20, 30, 40, 60, 120 min, the mixture was centrifuged at 5000× *g* for 5 min after incubation, the supernatant was collected and detected at 660 nm by spectrophotometer method, the adsorption amount was calculated by Equation (2); (**b**) is effect of adsorption temperature on adsorption rate of methylene blue, methylene blue solution was prepared with content of 50 mg/L, 0.15 g of ACH was placed in 100 mL of methylene blue solution and incubated at 20, 30, 40, 50, 60 °C for 20, 30, 40, 50, 60 min, the mixture was centrifuged at 5000× *g* for 5 min after incubation, the supernatant was collected and detected at 660 nm by spectrophotometer method, the adsorption amount was calculated by Equation (2); (**c**) is comparison results of different materials in different adsorption time on adsorption rate of methylene blue, methylene blue solution was prepared with content of 200 mg/L, 0.10 g of prepared starch grafting acrylic acid (S), α-CD grafting acrylic acid (B), acrylic acid grafting complex gel—ACH (C) and acrylic acid grafting α-CD/F127 (A) was placed in 100 mL of methylene blue solution, for A and C, it was incubated at 20 °C for 5, 10, 15, 20, 25, 30, 40, 60, 120 min, for B and S, it was incubated at 20 °C for 10, 20, 30, 40, 70, 100, 130 min, the mixture was centrifuged at 5000× *g* for 5 min after incubation, the supernatant was collected and detected at 660 nm by spectrophotometer method, the adsorption amount was calculated by Equation (2); (**d**) is the picture of ACH before and after adsorption of methylene blue.

Figure 3a shows that an increase in methylene-blue solution initial concentration results in a gradual increase in adsorption capacity. The initial adsorption efficiency is relatively high, which results because the adsorption sites of the gel are empty initially. With an increase in adsorption time, the adsorption sites become occupied and the adsorption efficiency slows down. The maximum adsorption capacity was 198.29 mg/g.

#### 2.4.2. Effect of Temperature on Gel Adsorption

With an increase in temperature, the adsorption rate of methylene blue also increased (Figure 3b). At 20 °C–40 °C, the adsorption rate of methylene blue on the gel increased rapidly, and reached a maximum of 99.3 ± 0.12% at 40 °C. This may result from the increase in temperature, which expanded the gel network. The adsorption process is endothermic and a higher temperature accompanies a higher adsorption efficiency.

#### 2.4.3. Comparative Experiments for Methylene-Blue Adsorption Properties

α-CD/Pluronic F127 hydrogels that were grafted with acrylation (A), α-CD that was grafted with acrylation (B), and starch that was grafted with acrylation (S) were prepared and added to 200 mg/L methylene-blue solution to observe the adsorption (Figure 3c). It can be seen from Figure 3 that in the control the curve slopes sharply upward in the 0–20 min range, was gentle from 20–60 min, and the adsorption rate slows down and becomes almost constant, which is consistent with the basic characteristics of liquid adsorption of a porous adsorbent. The adsorption of methylene blue for B and S tends to increase continuously. Before 40 min, the adsorption rates followed control > A > B > S. After 120 min, the system reached equilibrium, and the equilibrium adsorption capacity decreased as control > A > S > B. This may result because there were more active groups in the control than in A, S, and B. In the free radical polymerization with acrylic acid, more acrylic acid grafts on the control, which causes the number of carboxyl groups to increase. The rate of methylene-blue adsorption and the final saturation of the adsorption capacity of the control is larger than that of the hydrogels that are prepared by other methods.

### 2.5. Adsorption Isotherms of Methylene Blue by ACH

The adsorption of methylene blue was established and fitted with the isothermal adsorption equation of Langmuir and Freundlich [24,25], in which the adsorption equation of Langmuir was:

(1) $$\frac{C_{e}}{Q_{e}}{\;=\;Q}_{\max}{\;\times \;C}_{e}\;+\;\frac{1}{\left(Q_{\max}{\;\times \;K}_{L}\right)}$$

where *C_e_* is the concentration of methylene blue in solution when the adsorption equilibrium is reached (mg/L), Mg/g indicates the equilibrium adsorption capacity of the unit adsorbent when the adsorption equilibrium is reached *Q_e_* (mg/g), *Q_max_* represents the amount of saturated adsorption of the unit adsorbent (mg/g), and *K_L_* indicates the coefficient of Langmuir adsorption.

The Freundlich adsorption equation is:

(2) $${\mathrm{logQ}}_{e}\;=\;\frac{1}{n}{\mathrm{logC}}_{e}{\;+\;\mathrm{logK}}_{F}$$

where *C_e_* and *Q_e_* are the same as in Equation (1), *N* stands for the Freundlich constant, *K_F_* is the coefficient of Freundlich adsorption, *N* determines the shape of the Freundlich isotherm, and 1/n is between 0.1–0.5. When 1/n is greater than 2, the adsorbate is not easily adsorbed by the adsorbent.

**Figure 4:**
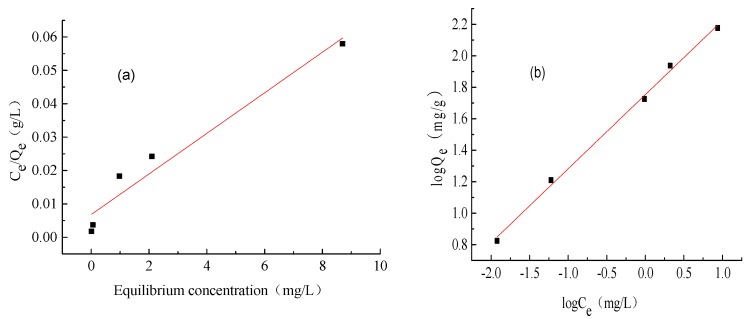
Linear Langmuir fitting (**a**) and Freundlich fitting (**b**) of adsorption isotherm.

The fitting diagram of the Freundlich and Langmuir adsorption isotherm results is shown in Figure 4. The relative parameters are given in Table 1.

The correlation coefficient *R*^2^ = 0.9542 for the Langmuir model and *R*^2^ = 0.997 for the Freundlich model, which indicates that the gel is more biased towards the Freundlich adsorption model.

The Langmuir model is suitable for describing the adsorption process with a homogeneous surface, which means that once adsorption occurs, the adsorption site will not be transferred. The Freundlich model is suitable for describing the adsorption of adsorbent with an uneven surface. Adsorbent can be adsorbed on multiple layers for a high-concentration adsorption. It can be deduced that the ACH adsorption process is comprised of methylene-blue diffusion to the gel surface, methylene blue spread on the gel surface and adsorption, and more methylene-blue spread into the inside of the gel.

### 2.6. Adsorption Kinetics of ACH

**Figure 5:**
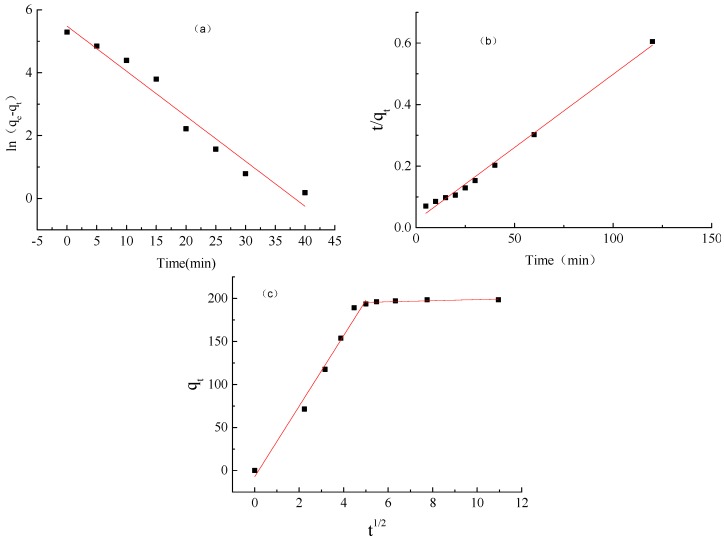
Quasi-first adsorption kinetics fitting (**a**); quasi-secondary adsorption kinetics fitting (**b**); and diffusion adsorption kinetic fitting (**c**) curves of methylene blue absorbed by ACH.

To understand the adsorption kinetics of methylene blue by ACH, the quasi-first-order kinetics, quasi-second-order kinetic equation, and the particle-diffusion model were used to fit the adsorption process. The fitted data are shown in Figure 5 and the fitting equations are shown in Table 2.

In quasi-first-order kinetics fitting, ln(*q_e_* − *q_t_*) is used on the *Y*-axis and t is used on the *X*-axis, where *q_e_* is the equilibrium adsorption capacity (mg/g) of the unit adsorbent after adsorption equilibrium and *q_t_* is the unit adsorption capacity at adsorbent time *t* (mg/g). Quasi-second-order kinetics used *t*/*q_t_* as the *Y*-axis and t as the *X*-axis, where *q_t_* is the unit adsorbent adsorption capacity at time *t*. The particle internal-diffusion model used *q_t_* as the *Y*-axis and *t*^½^ as the *X*-axis, where *q_t_* is the amount of adsorption at time *t* (mg/g) [26]. Table 2 shows that the *R*^2^ for the quasi-first-order kinetic equation is 0.9629, and that for the quasi-second-order kinetic equation is 0.9935.

The equilibrium concentration of methylene blue in the quasi-first-order kinetic model is 240.64 mg/L, but is 208.33 mg/L for the quasi-second-order kinetic model. The actual equilibrium concentration was 198.4 mg/L. The quasi-second-order kinetics model gives much closer data to the experimental value, which indicates that the process of methylene-blue adsorption is more appropriate as quasi-second-order kinetics. In accordance with the quasi-second-order kinetics, the chemisorption in the adsorption reaction is decisive in controlling adsorption, not diffusion, means the adsorption of methylene blue on the COO of acrylic acid was the decisive effect. Further, Figure 5c shows that two fitting lines exist in the diagram, which means external and internal mass-transfer resistance existed for methylene-blue diffusion in the gel.

### 2.7. Release Characteristics of ACHs

**Figure 6:**
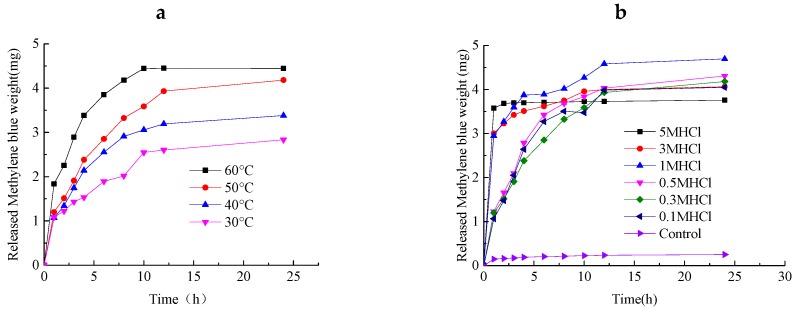
Effects of different conditions on methylene blue release. (**a**) is release amount of methylene blue in different time under different temperature, the release experiments were carried out as following steps: 1 g of ACH after adsorption of methylene blue was placed in 100 mL of 1 M HCL solution, the mixture was incubated at 30, 40, 50, 60 °C for 1, 2, 3, 4, 6, 8, 10, 12, 24 h, separately, the mixture was centrifuged at 2500× *g* for 5 min after incubation, the supernatant was collected and detected at 660 nm by spectrophotometer method; (**b**) is release amount of methylene blue in different time with different concentration of HCL added, the release experiments were carried out as following steps: 1 g of ACH after adsorption of methylene blue was placed in 100 mL of HCL solution with concentration of 0.1, 0.3, 0.5, 1, 3, 5 M, separately, the mixture was incubated at 30, 40, 50, 60 °C for 1, 2, 3, 4, 6, 8, 10, 12, 24 h, separately, the mixture was centrifuged at 2500× *g* for 5 min after incubation, the supernatant was collected and detected at 660 nm by spectrophotometer method.

The effects of temperature and hydrochloric-acid concentration on the release ratio of methylene blue were investigated as shown in Figure 6a. When the temperature is less than 60 °C, the release equilibrium is not reached within 24 h; the amount of methylene blue that released is 2.83, 3.38, and 4.18 mg at 30 °C, 40 °C, and 50 °C, respectively. The release equilibrium was reached at 60 °C after 24 h of release, and the amount of methylene blue that was released was 4.44 mg. The release rate of methylene blue increases gradually with an increase in temperature, and the amount released increased gradually.

Hydrochloric acid was added into the ACH-methylene blue adsorption system to observe the release process. Figure 6b shows that the release rate increases with an increase in hydrochloric acid concentration. When the concentration of hydrochloric acid is 5 M, the release rate of methylene blue is fastest, and the release equilibrium is reached at 2 h, but only 3.76 mg of methylene blue was released. The maximum amount of methylene blue that was released occurred with a 1-M hydrochloric acid addition, and the release equilibrium was reached at 12 h. A higher concentration of hydrochloric acid yielded a higher conversion of carboxylate (COO-) in the ACH to carboxyl (COOH), and the electrostatic attraction between the methylene blue molecules disappears. The methylene blue is readily removed from the hydrogel through internal diffusion. However, the effect of pH on the swelling of the gel shows that ACH is pH-sensitive. Under acidic conditions, the smaller pH yields a smaller ACH swelling degree. Less methylene blue is absorbed and released because of the gel shrinkage. ACH reuse for absorbed and released methylene blue shows that the removal rate of methylene blue was still 70.1% after three repeats when using 75 mg/L of methylene blue for adsorption.

## 3. Materials and Methods

### 3.1. Preparation of Citric Acid β-Cyclodextrin (CA-β-CD) Prepolymers

Citric acid (CA, 4.2 g), sodium hypophosphite (1.06 g), β-CD (11.35 g), and deionized water (6.81 g) (Sinopharm Group, Wuxi, China) were mixed and placed into a 100 °C oven for 2 h. The product was extracted with absolute ethanol (Sinopharm Group, Wuxi, China) for 6 h and dissolved in a small amount of deionized water. Acetone (Sinopharm Group, Wuxi, China) was added to precipitate the sample, which was filtered, and the procedure was repeated three times. The precipitate was dried in a 60 °C oven overnight.

### 3.2. Infrared Characterization of CA-β-CD

Samples were mixed with dry potassium bromide (Sinopharm Group, Wuxi, China) in a ratio of 1:50 to 1:100, ground, and pressed. Samples were heated under infrared light for 5 min to remove adsorbed moisture. The scan interval was 400–4000 cm^−1^, with 32 scans at a resolution of 4 cm^−1^.

### 3.3. Grafting CA-β-CD to CNC

A certain amount of CA-β-CD was dissolved in 10 mL deionized water. Sodium hypophosphite (0.1 g) was added and the pH was adjusted to 3.5 by using 0.1 M hydrochloric acid and sodium hydroxide solutions. CNC (1 g, Sinopharm Group, Wuxi, China) was added to the solution and stirred for 30 min in a 160 °C oil bath. After stirring for 30 min, the product was collected, washed three times with 60 °C water and dried in a vacuum oven at 60 °C to obtain CNC-β-CD.

### 3.4. Preparation of Composite CD Hydrogel (CCH)

CNC-β-CD of 0.5% *w*/*v* (0.025 g), 1% (*w*/*v*) (0.05 g), 1.5% (*w*/*v*) (0.075 g), and 2% (*w*/*v*) (0.1 g) was dispersed it in 3.5 mL deionized water; 0.35 g of the block copolymer Pluronic F127 (Sigma, St. Louis, MO, USA) was added; and the mixture was stirred for 24 h at room temperature to obtain a clear liquid. α-CD (0.485 g, Sigma, St. Louis, MO, USA) was dissolved in 1.5 mL deionized water and heated at 60 °C to obtain a clear liquid. α-CD solution was mixed with Pluronic F127 and CNC-β-CD solution, stirred for 2 min and sonicated for 5 min. CCH was formed after setting at room temperature to form a gel.

### 3.5. Acrylated Composite Hydrogels (ACH) Preparation

Based on the literature [27], 0, 0.5, 1, 1.5, and 2 g of freeze-dried CCH was weighed in 30 mL. Acrylic-acid solution (60%, 65%, 70%, 75%, 80%, 85%), 10 mL 0.01 g/mL *N*,*N*′-methylene bisacrylamide, and 10 mL 0.1 g/mL potassium persulfate were added to 50 mL deionized water. The mixtures were kept at 50 °C, 55 °C, 60 °C, 65 °C, and 70 °C to form gels. The resulting products were extracted with acetone and evacuated in a Soxhlet extractor (Sinopharm Group, Wuxi, China) for 12 h. The swelling ratio was calculated from:

(3) $$\mathrm{Swelling}\;\mathrm{ratio}\;(\%)=\frac{M_{E}{\;-\;M}_{0}}{M_{0}}$$

where M_E_ is the gel mass at equilibrium (g) and M_0_ is the gel mass before water adsorption (g).

### 3.6. Grafted Acrylic Acid with α-CD and Starch

A comparative experiment was carried out by grafting acrylic acid with α-CD and starch. α-CD (1 g) and starch were weighed and placed in 30 mL neutralized 80% acetic acid solution, respectively. Deionized water (50 mL), *N*,*N*′-methylenebisacrylamide (10 mL, 0.01 g/mL, Sinopharm Group, Wuxi, China), and potassium persulfate (10 mL, 0.1 g/mL) were added into the solution, and the mixtures were maintained at 60 °C to achieve gelatinization. The product was extracted with acetone as a solvent and evacuated in a Soxhlet extractor for 12 h.

### 3.7. Effect of pH on ACH Swelling Ratio

ACH (0.1 g,) was placed in a water solution with pH changed into 1, 3, 5, 7, 9, and 11 by HCL and NaOH (Sinopharm Group, Wuxi, China). After observed the stable volume of ACH gel, surface water was removed by using filter paper and the sediment was weighed. The swelling ratio was calculated by Equation (3).

### 3.8. Determination of ACH Adsorption Properties

Composite CD hydrogel that was grafted with acrylic acid (0.15 g) was added to 100 mL of 10, 50, 75, 150, and 300 mg/L methylene-blue solution and shaken at 20 °C, 30 °C, 40 °C, 50 °C and 60 °C for oscillation adsorption. The supernatant was collected after being centrifuged at 5000× *g* for 5 min and analyzed by spectrophotometer. The adsorption was calculated by Equation (4).

(4) $$q_{e}=\frac{V\;\times \;\left(C_{0}{\;-\;C}_{e}\right)}{M}\;$$

where *q_e_* is the adsorption capacity at equilibrium (mg/g); *V* is volume of solution (L); *C_e_* is concentration of methylene blue after equilibrium (mg/L); *C*_0_ is the initial concentration of methylene blue (mg/L); *M* is the weight of adsorbent (g).

### 3.9. ACH Release Properties

A hydrochloric acid solution with concentration of 0.1, 0.3, 0.5, 1, 3, and 5 M was prepared separately, then 1 g hydrogel that adsorbed with methylene blue was added to 100 mL hydrochloric acid solution, and incubate at 30 °C, 40 °C, 50 °C, and 60 °C at regular intervals of time, and the supernatant was collected after being centrifuged at 5000× *g* for 5 min and analyzed by spectrophotometer.

## 4. Conclusions

The introduction of acrylic acid into CCH could improve the swelling mechanism and adsorption properties of the gel. The ACH adsorption kinetics were investigated and the adsorption is based mainly on chemical and physical adsorption. It provides an idea to improve the mechanism property and adsorption of hydrogels, thus there is potential applications of this novel gel being used as water purification for removing organic pollutions. The release characters of this gel also provides a temperature- and pH-stimulation release gel, which can be used as smart gel for controlled release of drugs. More works need to do to extend the final application of ACH.

## Figures and Tables

**Table 1:** Langmuir isotherm and Freundlich isotherm parameters.

Equation	Equation	Saturated Adsorption Capacity Qmax (mg/g)	Adsorption Coefficient *K_L_*	*R*^2^
Langmuir	*C_e_*/*Q_e_* = 0.0061*C_e_* + 0.0068	163.93	0.897	0.9542
Freundlich	log*Q_e_* = 0.4708log*C_e_* + 1.7529	56.61	2.124	0.997

**Table 2:** Results of fitting dynamics equation.

Equation	Equation	Balance Concentration (mg/L)	*R*^2^
Quasi—first order kinetics equation	*y* = −0.1433*x* + 5.4833	240.64	0.9629
Quasi—second order kinetics equation	*y* = 0.0048*x* + 0.0227	208.33	0.9935

## References

[1] Yuan Y., Zhang L. (2010). Recent progress in the study of cyclodextrin-based environment sensitive hydrogel. Yao Xue Xue Bao.

[2] Li J., Harada A., Kamachi M. (1994). Sol–gel transition during inclusion complex formation between α-cyclodextrin and high molecular weight poly (ethylene glycol) s in aqueous solution. Polym. J..

[3] Zhao Y.-L., Stoddart J.F. (2009). Azobenzene-based light-responsive hydrogel system. Langmuir.

[4] Matsumoto S., Yamaguchi S., Ueno S., Komatsu H., Ikeda M., Ishizuka K., Tabata K.V., Aoki H., Ito S., Noji H. (2008). Photo Gel–Sol/Sol–Gel Transition and Its Patterning of a Supramolecular Hydrogel as Stimuli-Responsive Biomaterials. Chem.-A Eur. J..

[5] Kretschmann O., Choi S.W., Miyauchi M., Tomatsu I., Harada A., Ritter H. (2006). Switchable Hydrogels Obtained by Supramolecular Cross-Linking of Adamantyl-Containing LCST Copolymers with Cyclodextrin Dimers. Angew. Chem. Int. Ed..

[6] Van de Manakker F., van der Pot M., Vermonden T., van Nostrum C.F., Hennink W.E. (2008). Self-assembling hydrogels based on β-cyclodextrin/cholesterol inclusion complexes. Macromolecules.

[7] Van de Manakker F., Vermonden T., el Morabit N., van Nostrum C.F., Hennink W.E. (2008). Rheological behavior of self-assembling PEG-β-cyclodextrin/PEG-cholesterol hydrogels. Langmuir.

[8] Choi H.S., Ooya T., Huh K.M., Yui N. (2005). pH-triggered changes in assembling properties of β-cyclodextrin-conjugated poly(ε-lysine) complexes. Biomacromolecules.

[9] Weickenmeier M., Wenz G., Huff J. (1997). Association thickener by host guest interaction of a β-cyclodextrin polymer and a polymer with hydrophobic side-groups. Macromol. Rapid Commun..

[10] Daoud-Mahammed S., Grossiord J., Bergua T., Amiel C., Couvreur P., Gref R. (2008). Self-assembling cyclodextrin based hydrogels for the sustained delivery of hydrophobic drugs. J. Biomed. Mater. Res. Part A.

[11] Gref R., Amiel C., Molinard K., Daoud-Mahammed S., Sébille B., Gillet B., Beloeil J.C., Ringard C., Rosilio V., Poupaert J. (2006). New self-assembled nanogels based on host–guest interactions: Characterization and drug loading. J. Control. Release.

[12] Sangeetha N.M., Maitra U. (2005). Supramolecular gels: Functions and uses. Chem. Soc. Rev..

[13] Wang Z., Chen Y. (2007). Supramolecular hydrogels hybridized with single-walled carbon nanotubes. Macromolecules.

[14] Ma D., Zhang L.-M. (2008). Fabrication and modulation of magnetically supramolecular hydrogels. J. Phys. Chem. B.

[15] Martel B., Weltrowski M., Ruffin D., Morcellet M. (2002). Polycarboxylic acids as crosslinking agents for grafting cyclodextrins onto cotton and wool fabrics: Study of the process parameters. J. Appl. Polym. Sci..

[16] Lanthong P., Nuisin R., Kiatkamjornwong S. (2006). Graft copolymerization, characterization, and degradation of cassava starch-g-acrylamide/itaconic acid superabsorbents. Carbohydr. Polym..

[17] Athawale V., Lele V. (1998). Graft copolymerization onto starch. II. Grafting of acrylic acid and preparation of it’s hydrogels. Carbohydr. Polym..

[18] Chen J., Rong L., Lin H., Xiao R., Wu H. (2009). Radiation synthesis of pH-sensitive hydrogels from β-cyclodextrin-grafted PEG and acrylic acid for drug delivery. Mater. Chem. Phys..

[19] Ahmed M.J. (2016). Application of agricultural based activated carbons by microwave and conventional activations for basic dye adsorption. J. Environ. Chem. Eng..

[20] Hiraku Y., Goto H., Kohno M., Kawanishi S., Murata M. (2014). Metal-mediated oxidative DNA damage induced by methylene blue. Biochim. Biophys. Acta (BBA)-Gener. Subj..

[21] Lo J.C., Darracq M.A., Clark R.F. (2014). A review of methylene blue treatment for cardiovascular collapse. J. Emerg. Med..

[22] Rafatullah M., Sulaiman O., Hashim R., Ahmad A. (2010). Adsorption of methylene blue on low-cost adsorbents: A review. J. Hazard. Mater..

[23] Foo K., Hameed B.H. (2010). Insights into the modeling of adsorption isotherm systems. Chem. Eng. J..

[24] Redlich O., Peterson D.L. (1959). A useful adsorption isotherm. J. Phys. Chem..

[25] Shuchuan P., Shisheng W., Tianhu C., Shaotong J., Chuanhui H. (2006). Adsorption kinetics of methylene blue from aqueous solutions onto palygorskite. Acta Geol. Sin. (Engl. Ed.).

[26] Hu J., Zheng S., Xu X. (2012). Dual stimuli responsive poly (*N*-isopropylacrylamide-co-acrylic acid) hydrogels based on a β-cyclodextrin crosslinker: Synthesis, properties, and controlled protein release. J. Polym. Res..

[27] Si H., Li B., Wang T., Lin L., Xu Z. (2013). Preparation of cyclodextrin grafting wood flour and investigation of the release characteristics of eugenol. Wood Sci. Technol..

